# Anti-Inflammatory Therapy in Coronary Artery Disease: Where Do We Stand?

**DOI:** 10.31083/j.rcm2401010

**Published:** 2023-01-04

**Authors:** Jelena Rakocevic, Milan Dobric, Milica Labudovic Borovic, Katarina Milutinovic, Sanela Milenkovic, Miloje Tomasevic

**Affiliations:** ^1^Institute of Histology and Embryology “Aleksandar Đ. Kostić”, Faculty of Medicine, University of Belgrade, 11000 Belgrade, Serbia; ^2^Institute for Cardiovascular Diseases “Dedinje”, 11000 Belgrade, Serbia; ^3^Faculty of Medicine, University of Belgrade, 11000 Belgrade, Serbia; ^4^Faculty of Pharmacy, University of Belgrade, 11000 Belgrade, Serbia; ^5^Department of Internal Medicine, Faculty of Medical Sciences, University of Kragujevac, 34000 Kragujevac, Serbia; ^6^Cardiology Clinic, University Clinical Center of Serbia, 11000 Belgrade, Serbia

**Keywords:** coronary artery disease, inflammation, anti-inflammatory therapy, CRP, IL-6, IL-1β, canakinumab, tocilizumab, colchicine

## Abstract

Inflammation plays an important role in all stages of atherosclerosis — from endothelial dysfunction, to formation of fatty streaks and atherosclerotic plaque, and its progression to serious complications, such as atherosclerotic plaque rupture. Although dyslipidemia is a key driver of atherosclerosis, pathogenesis of atherosclerosis is now considered interplay between cholesterol and inflammation, with the significant role of the immune system and immune cells. Despite modern therapeutic approaches in primary and secondary cardiovascular prevention, cardiovascular diseases remain the leading cause of mortality worldwide. In order to reduce residual cardiovascular risk, despite the guidelines-guided optimal medical therapy, novel therapeutic strategies are needed for prevention and management of coronary artery disease. One of the innovative and promising approaches in atherosclerotic cardiovascular disease might be inflammation-targeted therapy. Numerous experimental and clinical studies are seeking into metabolic pathways underlying atherosclerosis, in order to find the most suitable pathway and inflammatory marker/s that should be the target for anti-inflammatory therapy. Many anti-inflammatory drugs have been tested, from the well-known broad range anti-inflammatory agents, such as colchicine, allopurinol and methotrexate, to targeted monoclonal antibodies specifically inhibiting a molecule included in inflammatory pathway, such as canakinumab and tocilizumab. To date, there are no approved anti-inflammatory agents specifically indicated for silencing inflammation in patients with coronary artery disease. The most promising results came from the studies which tested colchicine, and studies where the inflammatory-target was NOD-, LRR- and pyrin domain-containing protein 3 (NLRP3) inflammasome/interleukin-1 beta (IL-1β)/interleukin-6 (IL-6)/C-reactive protein (CRP) pathway. A growing body of evidence, along with the ongoing clinical studies, suggest that the anti-inflammatory therapy might become an additional strategy in treating atherosclerotic cardiovascular disease. Herein we present an overview of the role of inflammation in atherosclerosis, the most important inflammatory markers chosen as targets of anti-inflammatory therapy, along with the critical review of the major clinical trials which tested non-targeted and targeted anti-inflammatory drugs in patients with atherosclerotic cardiovascular disease.

## 1. Introduction

Atherosclerosis has long been considered the “lipid storage disease” which develops due to mechanical accumulation of cholesterol in the subintimal space of the arteries. Therefore, it was expected that the aggressive pharmacological approach in treating hypercholesterolemia would reduce the prevalence, or even eliminate coronary artery disease (CAD). Intensive reduction of low-density lipoproteins (LDL)-cholesterol can be achieved by high-intensity statins, addition of ezetimibe and powerful hypolipidemic agents - PCSK9 (Proprotein Convertase Subtilisin/Kexin type 9) inhibitors, such as evolocumab and alirocumab [1]. Despite the large reduction in LDL-cholesterol, followed by the lower cardiovascular events, many patients are left with residual cardiovascular risk [2].

Although Rudolf Virchow recognized the role of inflammatory cells in atherosclerosis more than 100 years ago, the significant role of inflammation in atherosclerosis became apparent and recognized over the last 20 years [3]. Common cardiovascular risk factors, such as hypertension, smoking, diabetes, and insulin resistance, often lead to chronic inflammation and therefore can partially explain residual cardiovascular risk.

Experimental and clinical studies have confirmed that the inflammation is incorporated in all stages of atherogenesis, starting from endothelial dysfunction and accumulation of foam cells, followed by the formation of fatty streaks and fibrous plaques, and finally taking part in severe complications, such as plaque thrombosis [4, 5]. Therefore, pathogenesis of atherosclerosis is now considered interplay between cholesterol and inflammation, with the significant role of the immune system and immune cells.

Despite modern therapeutic approaches and aggressive measures of secondary prevention, cardiovascular diseases remain the leading cause of mortality worldwide. Novel strategies are needed for prevention and management of coronary artery disease. In that light, inflammation-targeted therapy emerged as an innovative and promising approach in atherosclerotic cardiovascular disease.

In this narrative review we will summarize key inflammatory targets tested in clinical settings, along with the critical analysis of success or failure of major clinical trials which explored the anti-inflammatory approach in coronary artery disease.

## 2. Inflammation in Atherosclerosis

Over the past two decades atherosclerosis evolved from a simple lipid-storage disease to inflammatory and immune-mediated disease, primarily localized in the intima, the inner layer of the arterial wall [1]. Inflammation plays an important role at all stages of atherosclerosis, from early intimal lesion to the development of complications. Many factors can trigger the development of atherosclerosis. Among the most frequent triggers are oxidized LDL. The early phases of atherosclerosis are characterized by endothelial dysfunction and higher endothelial permeability. Greater permeability allows the passage of plasma constituents, including LDL, from blood to the intimal layer. According to the oxidation hypothesis, LDL particles localized in the intimal layer undergo oxidative modification [1]. Modified lipid components propel the next steps of atherosclerosis: induce the expression of adhesion molecules, and the secretion of inflammatory cytokines in macrophages and other cell types. Therefore, dyslipidemia is considered one of the most potent risk factors for the development of atherosclerosis. Other important risk factors include hypertension, hyperglycaemia, smoking, obesity, infection, hyperhomocysteinemia *etc*. [6, 7].

Whether these triggers act individually, or are present at the same time, atherosclerosis can be divided into several stages. Endothelial dysfunction and increased lipid accumulation in the subendothelial layer are considered the cornerstone for the formation and progression of atherosclerotic plaque. Endothelial cells normally express numerous surface molecules which act as receptors, and at the same time facilitate their identification and distinction from other cell types (so-called endothelial cell markers) [8]. Due to endothelial dysfunction and lipid accumulation, endothelial cells show increased expression of adhesion cell molecules at their surface, such as intercellular adhesion molecule-1 (ICAM-1) and vascular cell adhesion molecule-1 (VCAM-1) [9]. Under the normal circumstances, leukocytes do not adhere to vascular endothelium. However, overexpression of the endothelial adhesion molecules promotes binding of leukocytes to the lining endothelium. Once firmly attached to the endothelium, leukocytes transmigrate into the subendothelial space [10].

When monocytes transmigrate endothelial layer and reside in the subintimal layer, they are differentiated into macrophages. Macrophages are capable of engulfing oxidized LDL, and are transformed into foam cells. At the same time, inflammation is augmenting, with further synthesis and release of proinflammatory molecules, recruitment of macrophages, activated T and B cells, their release of cytokines and chemokines, and further accumulation of lipids [4]. This vicious circle can be attenuated; smooth muscle cells migrated from the arterial medial layer can secrete collagen and other extracellular matrix components. That way, the fibrous cap covers the lipid-inflammatory core and stabilizes the atherosclerotic plaque. On the other hand, macrophages secrete specific type of enzymes, matrix metalloproteinases, which can digest collagen and other constituents of extracellular matrix, leading to plaque destabilization. The outcome and clinical manifestations of atherosclerosis rely on the balance between these two processes.

## 3. Why is C-Reactive Protein Not An Inflammatory Target?

C-reactive protein (CRP) is an acute phase reactant made in the liver as a response to inflammatory cytokines, particularly interleukin-6 (IL-6). CRP has several advantages over other inflammatory biomarkers; CRP is an inexpensive biomarker with a long half-life, whose levels are stable over longer periods of time [11]. Numerous clinical studies have confirmed that CRP is a strong and independent predictor of cardiovascular events in patients with and without known cardiovascular disease [12, 13, 14]. Therefore, higher CRP values may point to the higher risk of future adverse cardiovascular events. Despite these results, CRP is not considered an adequate inflammatory therapy target in coronary artery disease.

CRP is involved in almost all processes of atherosclerosis. This biomarker is shown to upregulate the expression of adhesion molecules on endothelial cells [15], and promotes recruitment of monocytes and their transformation into foam cells [16]. CRP is also involved in pathways of complement activation [17], and may interfere with plaque destabilization by inducing endothelial cell apoptosis and synthesis of matrix metalloproteinases [18].

On the other hand, CRP appears not to have a causal role in atherosclerosis. Although it is present in all stages of atherosclerotic plaque, CRP is considered a mediator of atherosclerosis. This is supported by the results of a large Mendelian randomization study where Elliott *et al*. [19] evaluated the polymorphism in genetic loci strongly associated with CRP levels. Authors analyzed polymorphism in 5 genetic loci which showed strong association with CRP. It was observed that analyzed variants in the CRP locus showed no association with coronary artery disease, confirming the mediating, rather than causal role of CRP in coronary artery disease. These results were further supported by the study of Zacho *et al*. [20], who additionally evaluated the risk of ischemic cerebrovascular disease. Polymorphism in CRP gene was analyzed in more than 10,000 people from the general population. Elevated levels of CRP >3 mg/L were associated with higher risk of ischemic heart disease and ischemic cerebrovascular disease. Interestingly, genetic polymorphisms in CRP gene associated with significant increase in CRP, were not associated with higher risk of ischemic vascular disease. Taken together, it appears that CRP polymorphism related to increased CRP values is not associated with the risk of ischemic vascular disease.

After the results of JUPITER study were published, CRP again gained clinical attention in patients with intermediate or uncertain levels of cardiovascular risk [21]. JUPITER (Justification for the Use of Statins in Primary Prevention: an Intervention Trial Evaluating Rosuvastatin) study included an apparently healthy population with no known cardiovascular disease. The goal of this study was to explore cardioprotective effects of statins in primary prevention. Included participants did not have hyperlipidemia; they did have elevated levels of LDL-cholesterol, but not elevated enough for prescribing statins per current guidelines (<3.4 mmol/L). On the other hand, all included participants had evidence of chronic inflammation with high sensitive CRP (hsCRP) ≥2 mg/L. Patients were randomly assigned to receive either rosuvastatin (20 mg daily), or placebo.

This study included almost 18,000 participants, with 5-year follow-up during which adverse cardiovascular events were recorded (composite of myocardial infarction, stroke, arterial revascularization, hospitalization for unstable angina, or death from cardiovascular causes) [21]. Average hsCRP values were similar in both groups (approximately 4.3 mg/L). Superiority of rosuvastatin was observed after a median of 1.9 years of follow-up, and therefore the study was terminated earlier than planned. Overall, rosuvastatin significantly reduced the risk of future cardiovascular events by 44% compared to placebo (hazard ratio (HR) 0.56; 95% confidence interval (CI) 0.46–0.69, *p *< 0.00001). These results were considered a breakthrough in clinical practice, underlying the importance of statins in primary cardiovascular prevention. This was particularly important for patients with intermediate or unclear cardiovascular risk who were not candidates for statin therapy. In these patients, CRP levels may point to the ones that would clinically benefit from statins.

But thorough insight into JUPITER results called for caution [22]. Hazard ratio of 0.56 translates into 44% relative risk reduction, which is an impressive lowering of cardiovascular risk. On the other hand, numbers appear a lot smaller when looking at the absolute rates and absolute risk reduction. Rates of cardiovascular adverse events were 0.77% per year in the rosuvastatin-group, and 1.37% per year in the placebo arm. Therefore, the absolute risk reduction for future cardiovascular events was only 0.59% per year. It should be mentioned that the composite of cardiovascular events encompassed events of different clinical severity — from unstable angina and arterial revascularization, to cardiovascular death.

JUPITER results raised another important question: did beneficial cardiovascular effects of rosuvastatin result from its cholesterol-lowering ability, or its anti-inflammatory effect? As previously mentioned, JUPITER study included patients whose LDL-cholesterol levels did not require statin therapy. Therefore, large variability in LDL-cholesterol reduction among participants did not come as a surprise. Interestingly, participants with the greatest reduction in LDL-cholesterol achieved the greatest reduction in cardiovascular adverse events; reduction of LDL-cholesterol for ≥50% was associated with 59% relative risk reduction in cardiovascular events, while patients with no reduction in LDL-cholesterol had 14% lower risk of adverse events [20].

Despite the given results, measurement of CRP levels did not find its way into European guidelines on cardiovascular prevention published in 2021, since it had limited additional value and did not improve risk prediction [23]. However, further research in targeted anti-inflammatory therapy in coronary artery disease shifted upstream from CRP, with encouraging and promising results.

## 4. Moving Upstream from CRP: IL-6, IL-1β and NOD-, LRR- and pyrin domain-containing protein 3 (NLRP3) Inflammasome

Exploring molecular pathways underlying atherosclerosis, interest in inflammatory biomarkers and targeted therapy has shifted upstream from CRP. In the previous several years, IL-1β and IL-6, two cytokines upstream from CRP, emerged as key players in atherosclerosis.

**Fig. 1.**
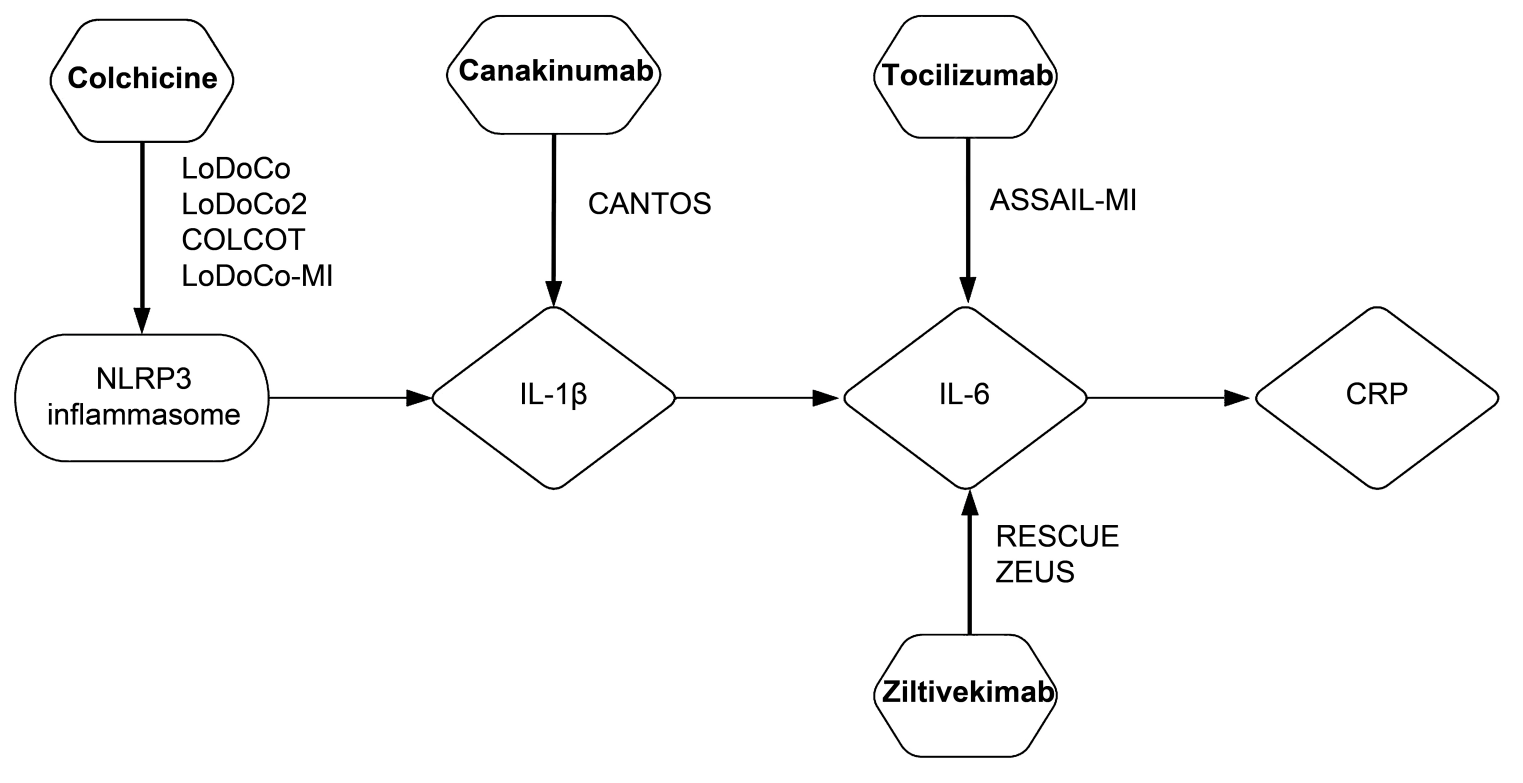
**NLRP3 inflammasome/IL-1β/IL-6/CRP inflammation pathway with the key experimental anti-inflammatory drugs tested in clinical trials**. NLRP3 inflammasome (left), an intracellular multiprotein complex of macrophages, transforms pro-IL-1β into its active form IL-1β. Colchicine primarily inhibits microtubules, which are important part of NLRP3 inflammasome, and was tested in LoDoCo, LoDoCo2, COLCOT, and LoDoCo-MI trials. IL-1β (center-left part of the figure) is an proinflammatory cytokine that promotes plaque instability and rupture, and stimulates production of IL-6. This pro-inflammatory molecule can be inhibited by canakinumab, monoclonal antibody, that was tested in CANTOS trial. IL-6 (center-left part of the figure) is another proinflammatory cytokine important for initiation and progression of atherosclerosis. This cytokine can be inhibited in two ways: by tocilizumab (monoclonal antibody which competitively binds to IL-6 receptors, tested in ASSAIL-MI trial), and by ziltivekimab (monoclonal antibody that directly targets IL-6 ligand, tested in RESCUE and ZEUS trials). CRP (right), final product of this pathway. NLRP3, NOD-, LRR- and pyrin domain-containing protein 3; IL-1β, interleukin-1 beta; IL-6, interleukin-6; CRP, C-reactive protein; LoDoCo, Low Dose Colchicine for CVD Prevention; COLCOT, Colchicine Cardiovascular Outcomes Trial; LoDoCo-MI, Low Dose Colchicine after Myocardial Infarction; ASSAIL-MI, ASSessing the Effect of Anti-IL-6 Treatment in Myocardial Infarction; RESCUE, Trial to Evaluate Reduction in Inflammation in Patients With Advanced Chronic Renal Disease Utilizing Antibody Mediated IL-6 Inhibition; ZEUS, Ziltivekimab Cardiovascular Outcomes Study.

Important step during inflammation is the formation of NLRP3 inflammasome in macrophages [24]. NLRP3 inflammasome is a complex intracellular multiprotein, a sensor which leads to caspase 1-dependent release of proinflammatory cytokines IL-1β and IL-18 from their precursors pro-IL-1β and pro-IL-18, respectively [25]. Release of these cytokines activates additional inflammatory cells, leading to the IL-6 synthesis. IL-6 is the main protein upstream of CRP, and therefore completes the NLRP3 inflammasome/IL-1β/IL-6/CRP inflammatory pathway (Fig. 1).

Association between atherosclerotic cardiovascular disease and inflammatory markers, such as IL-6 and IL-1β was confirmed in clinical studies which included apparently healthy men and women [13, 26]. While CRP found its position in everyday clinical practice, biomarkers upstream from CRP did not translate from bench to bedside. This could be explained by their instability in the blood samples, lack of assay standardization and variability of results, low availability, and the price of assays.

## 5. Targeting IL-1β: Canakinumab

Relying on the higher CRP values, the results of JUPITER trial pointed to the subpopulation of patients which could benefit from statin therapy, although it was not indicated [21]. However, it was unclear whether clinical benefit of statin resulted from its lipid-lowering or anti-inflammatory effect. The only way to prove inflammatory hypothesis and anti-inflammatory approach in coronary artery disease was to test an agent that exclusively inhibited inflammation.

IL-1 is an important inflammatory cytokine upstream from IL-6, with IL-1β being its main circulating form [27]. Experimental studies showed that atherosclerosis-prone mice deficient in IL-1β have smaller atherosclerotic plaques, and that atherosclerotic plaque increases in mice exposed to IL-1β [28]. As previously mentioned, activated NLRP3 inflammasome transforms pro-IL-1β into its active form IL-1β, which further magnifies inflammation by following mechanisms: first, IL-1β promotes plaque instability and rupture, and second, IL-1β stimulates IL-6 synthesis, that acts as a signal for CRP synthesis in the liver [29] (Fig. 1).

Therefore, CANTOS (Canakinumab Anti-inflammatory Thrombosis Outcome Study) study was designed to confirm the independent role of inflammation in atherosclerosis, and to explore inhibition of IL-1β as a therapeutic option for coronary artery disease [30]. This was a proof-of-concept study which tested canakinumab, a human monoclonal antibody targeting IL-1β. Canakinumab has an entirely anti-inflammatory effect, with no effect on blood lipid levels. CANTOS was a secondary prevention study, which included 10,061 participants with prior myocardial infarction and CRP values ≥2 mg/L. Along with standard therapy, patients were randomized to receive canakinumab (subcutaneously, in three available doses — 50 mg, 150 mg or 200 mg, every 3 months), or placebo. As expected, canakinumab had no effect on LDL-cholesterol levels, but significantly reduced the values of CRP [30].

Clinical efficacy of canakinumab was evaluated through the occurrence of nonfatal myocardial infarction, nonfatal stroke, or cardiovascular death. After the average follow-up of 3.7 years, canakinumab at a dose of 150 mg reduced the rate of major adverse cardiovascular events (MACE) by 15% when compared to placebo (HR 0.85, 95% CI, 0.74–0.98) [30]. This effect was primarily driven by the reduction in myocardial infarction by 24% (HR 0.76, 95% CI, 0.62–0.92), with no significant effect on cardiovascular mortality. Additionally, canakinumab led to 17% risk reduction in hospitalization due to unstable angina that led to urgent revascularization.

Secondary analysis of CANTOS trial showed that canakinumab efficacy depended on the degree of inflammation reduction. Therefore, in participants who achieved CRP <2 mg/L after the first dose of canakinumab, MACE was reduced by 25% [31]. Conversely, in participants whose on-treatment CRP values were ≥2 mg/L, MACE risk was insignificantly reduced by 5%. Moreover, in participants who achieved CRP values <2 mg/L, risk of cardiovascular mortality was reduced by 31%, along with the reduction of all-cause mortality by 31%.

CANTOS study results were the most convincing evidence so far regarding the efficacy of anti-inflammatory therapy in atherosclerotic coronary artery disease. However, canakinumab was associated with significantly higher incidence of fatal infections and sepsis compared to placebo [30]. Also, canakinumab led to reduced platelet values, with no change in bleeding risk. Therefore, moderate cardioprotective role of canakinumab in coronary artery disease, accompanied by the risk of serious infections, significant cost of monoclonal antibody therapy and regulatory obstacles in the drug approval, resulted in cessation of further investigation and seeking approval for canakinumab by the drug developer.

After the study was finished, *post hoc* analysis revealed one interesting fact; despite the inhibition of IL-1β by canakinumab, patients still had residual inflammatory risk associated with IL-18 and IL-6 [32].

## 6. Targeting IL-6 Receptor: Tocilizumab

IL-6 is the main cytokine upstream from CRP [29]. Since CRP mainly reflects the mechanism underlying atherosclerosis, IL-6 is causally involved in this process. Experimental studies have shown that IL-6 contributes to initiation and progression of atherosclerotic plaque [33]. Ridker *et al*. [13] observed that higher levels of IL-6 in the healthy population are associated with elevated risk of myocardial infarction. This was supported by clinical evidence, where IL-6 proved to be an independent predictor of mortality in patients with acute coronary syndrome [34].

Tocilizumab is another monoclonal antibody which blocks the effects of IL-6 by competitively binding to IL-6 receptor. This drug is indicated in the therapy of rheumatoid arthritis, systemic juvenile idiopathic arthritis, giant cell arteritis, and systemic sclerosis-associated interstitial lung disease [35]. With its ability to inhibit CAR T cell-induced severe or life-threatening cytokine release syndrome, tocilizumab is approved for the treatment of COVID-19 in hospitalized adults and children [36]. Tocilizumab could reduce initiation and progression of atherosclerosis [37], and stabilize atherosclerotic plaque [38]. Additionally, tocilizumab may interfere with ischemic-reperfusion injury [39] and left ventricle remodeling [40]. Administration of single dose of tocilizumab in patients with non-ST-elevation acute myocardial infarction (NSTEMI) prior to coronary angiography resulted in lower levels of high sensitivity CRP (hsCRP) and lower high sensitivity troponin T (hsTnT) during the first three days of hospitalization [41]. This study indirectly showed that tocilizumab is capable of silencing inflammation and protecting cardiomyocytes in NSTEMI patients.

However, tocilizumab has not been tested in large randomized clinical trial evaluating hard clinical endpoints. ASSAIL-MI (*ASSessing the Effect of Anti-IL-6 Treatment in Myocardial Infarction*) was a randomized placebo-controlled study included 199 patients with the first ST-elevation myocardial infarction (STEMI) presenting within 6 hours of symptom onset [42]. Prior to percutaneous coronary intervention (PCI), patients were randomized to receive tocilizumab (in a dose of 280 mg as a single infusion), or placebo. The two interventions were compared by the myocardial salvage index, the measurement to which myocardium recovers after reperfusion. Myocardial salvage index was measured by cardiac magnetic resonance (CMR) 3–7 days after PCI. This small, proof-of-concept study showed that tocilizumab is associated with less irreversibly damaged myocardium compared to placebo administration; myocardial salvage index was significantly higher in tocilizumab-group compared to placebo group (69.3% vs. 63.6%, *p* = 0.04). Although patients receiving tocilizumab had less extensive microvascular obstruction, that did not reflect on infarct size measured 6 months after PCI. Final infarct size (percentage of the left ventricle mass) was similar in tocilizumab- and placebo-group (7.2% and 9.1%, respectively, *p* = 0.08). Study limitation was that study patients had relatively small-size myocardial infarction, which hindered the difference in outcomes. However, ASSAIL-MI study demonstrated a signal towards the reduction of infarct size with tocilizumab [42].

As expected, tocilizumab was associated with significant reduction in CRP values compared to placebo, although there was no difference in TnT values. Authors noted that tocilizumab had a more pronounced effect on patients presenting within 3 hours of symptom onset [42]. Limited 6-month follow-up showed no safety signal of tocilizumab, but larger studies are needed to confirm its cardioprotective role and establish its safety.

However, the possibility that long-term IL-6 inhibition may lead to up-regulation of lipoprotein B and increase in LDL-cholesterol, potentially limits its prolonged administration [43]. Tocilizumab effect might have been different in the population of patients with larger myocardial infarction, or with higher risk of ischemia-reperfusion injury. Additionally, ASSAIL-MI study explored the cardioprotective role of tocilizumab, with its primary impact of myocardium. Whether tocilizumab acts on atherosclerosis and to which extent remains elusive.

## 7. Targeting IL-6: Ziltivekimab

Ziltivekimab inhibits IL-6 differently from tocilizumab; instead of blocking IL-6 receptor, ziltivekimab is a human monoclonal antibody directly targeting IL-6 ligand. The anti-inflammatory effect of ziltivekimab was explored in RESCUE (Trial to Evaluate Reduction in Inflammation in Patients With Advanced Chronic Renal Disease Utilizing Antibody Mediated IL-6 Inhibition) clinical study which included participants at high cardiovascular risk [44]. This was a phase II study which enrolled 246 patients with moderate to severe chronic kidney disease, and hsCRP values ≥2 mg/L. Patients were randomized into 4 equal groups; three groups were receiving ziltivekimab as subcutaneous injections in three different doses (7.5 mg, 15 mg, or 30 mg) every 4 weeks up to 24 weeks. The fourth group received a matching placebo.

Anti-inflammatory effect of ziltivekimab was evaluated after the first 12 weeks of treatment, with the change in hsCRP values compared to baseline levels. Results showed dose-dependent anti-inflammatory effect of ziltivekimab; average hsCRP levels were reduced by 77% in the 7.5 mg-group, 88% in the 15 mg-group, and 92% in the 30 mg-group. At the same time, reduction of hsCRP in the placebo group was only 4% [44]. Likewise, ziltivekimab was well tolerated by patients.

Future investigation of ziltivekimab includes a large phase III ZEUS (Ziltivekimab Cardiovascular Outcomes Study) trial [45]. This study will include 6200 patients with atherosclerotic cardiovascular disease, chronic kidney disease (stage III–IV) and elevated CRP ≥2 mg/L. Hopefully the results of ZEUS study will indicate whether ziltivekimab succeeded in reducing MACE in these patients.

## 8. Methotrexate: Neutral Anti-Inflammatory Effect in Coronary Artery Disease

Methotrexate is a well-known anti-inflammatory and immunomodulatory drug indicated in the treatment of rheumatoid arthritis and other autoimmune diseases [46]. Unlike the targeted effect of monoclonal antibodies, methotrexate demonstrates broad-spectrum anti-inflammatory effect.

Potential cardioprotective effect of methotrexate was initially recorded in observational studies that included patients with psoriasis and rheumatoid arthritis [47, 48]. Danish nationwide study of patients with severe psoriasis showed that methotrexate treatment was associated with 35% risk reduction in MACE [47]. Results from the QUEST-RA study confirmed the similar results in patients with rheumatoid arthritis; with methotrexate the risk of cardiovascular morbidity was reduced by 15% [48].

Anti-inflammatory effect of methotrexate in stable patients with coronary artery disease was tested in CIRT (Cardiovascular Inflammation Reduction Trial) trial [49]. This trial ran parallel with CANTOS trial, and was led by the same group of authors. Unlike the positive results with canakinumab in CANTOS trial [30], methotrexate showed a neutral anti-inflammatory effect in CIRT trial.

CIRT trial included 4786 patients with previous myocardial infarction and multivessel coronary artery disease, who had additional metabolic and vascular burden with either type 2 diabetes or metabolic syndrome [49]. Majority of patients were already on statin therapy (86%), with average LDL-cholesterol levels of 1.76 mmol/L, and average CRP concentration of 1.5 mg/L. Therefore, inclusion criterion was not the existing systemic inflammation with elevated CRP level, unlike the patients in CANTOS study, which could partially explain opposite results. Participants were randomized to receive low-dose methotrexate at a target dose of 15–20 mg weekly, or placebo.

The trial was stopped after a median follow-up of 2.3 years for futility. Methotrexate failed to reduce the risk of nonfatal myocardial infarction, nonfatal stroke, or cardiovascular death (HR 1.01; 95% CI 0.82–1.25) [49]. Before the final results were published, authors wanted to provide greater statistical power with small sample size by expanding the primary endpoint. Initial primary endpoint was expanded with hospitalization for unstable angina that led to urgent revascularization. Even then, there was no reduction in the expanded primary endpoint (HR 0.96; 95% CI 0.79–1.16), and the trial was stopped for futility. At the same time, methotrexate showed no effect on CRP, IL-6, or IL-1β. As far as safety is concerned, methotrexate was associated with elevation in liver-enzyme levels, modest leukopenia, and significant increase in non-basal skin cancer (31 vs. 10; rate ratio 3.08, *p* = 0.002).

Therefore, neutral results of the CIRT trial excluded methotrexate, generic and inexpensive anti-inflammatory drug, as an option for treating residual inflammatory risk in coronary artery disease. There were several explanations of neutral CIRT results. First, CIRT study included patients without elevated CRP concentration, unlike CANTOS trial. In the setting of low or no residual inflammation, greater anti-inflammatory effect could not be expected. Second, when the CANTOS and CIRT studies were designed, there was no firm evidence on which inflammatory pathway was an essential therapy target. With the results of CANTOS trial, it became clear the IL-1β/IL-6/CRP pathway should be the target.

## 9. Colchicine: Repurposing the Old Drug

Colchicine is so far the only non-targeted broad-spectrum anti-inflammatory drug which showed cardioprotective effects in patients with atherosclerotic cardiovascular disease [50, 51]. This drug is indicated for prophylaxis and the treatment of gout flares, as well as in patients with familial Mediterranean fever [52]. Colchicine has been present in everyday clinical practice for decades, it is inexpensive, available and safe. Similarly to methotrexate, it was observed that patients with gout treated with colchicine had significantly lower prevalence of myocardial infarction than patients not taking colchicine (1.2% vs. 2.6%, *p* = 0.03) [53]. There was also a non-significant trend towards lower mortality and lower CRP levels among patients from colchicine-group. These findings were confirmed by Solomon *et al*. [54] in a retrospective analysis of gout patients. Two matching cohorts were formed, with one group being colchicine users, and the other group of non-users. After a median follow-up of 16.5 months, patients from the colchicine-group had 49% lower risk of composite myocardial infarction, stroke, or transitory ischemic attack.

Anti-inflammatory effect of colchicine in stable coronary disease was tested in a small, pilot study [55]. This study included 200 patients with hsCRP values ≥2 mg/L, despite background aspirin and statin therapy. Low-dose colchicine (0.5 mg twice daily) reduced CRP levels by an average 60% in patients with stable coronary disease, independently of aspirin and statin therapy.

Following study, LoDoCo (Low Dose Colchicine for CVD Prevention), included even more patients with stable coronary disease (n = 532) who were already on optimal medical therapy [50]. Patients were randomized into two groups — colchicine (0.5 mg daily) or matching placebo. This time the efficacy of colchicine was measured by composite of acute coronary syndrome, out-of-hospital cardiac arrest, or noncardioembolic ischemic stroke. Colchicine proved a clinical translation of its anti-inflammatory effect; after the median 3-year follow-up there was 67% risk reduction in primary outcome events (HR 0.33; 95% CI 0.18–0.59, *p *< 0.001). This reduction was primarily driven by the reduction in acute coronary syndrome (HR 0.33; 95% CI 0.18–0.63, *p *<0.001). Out-of-hospital cardiac arrest and noncardioembolic ischemic stroke were infrequent events, but with lower frequency in colchicine-group [50].

Positive results of LoDoCo trial led to the next trial, LoDoCo2, which included 5522 patients with chronic coronary disease [51]. All included patients clinically stable, and coronary artery disease was confirmed by coronary angiography or computed tomography angiography. Following randomization 2762 patients were assigned to colchicine (0.5 mg once daily), while 2760 patients received placebo. Cardioprotective effect of colchicine was evaluated by the occurrence of MACE (cardiovascular death, spontaneous myocardial infarction, ischemic stroke, or ischemia-driven coronary revascularization) during 28.6 months of follow-up. Long-term administration of colchicine was associated with 31% risk reduction in MACE compared to placebo (HR 0.69; 95% CI 0.57–0.83, *p *< 0.001). MACE occurred in 6.8% patients from colchicine group, and 9.6% patients from placebo-group [51].

Looking at the individual components of primary outcome, MACE risk reduction was primarily driven by 30% lower risk of spontaneous myocardial infarction, and 25% risk reduction in ischemia-driven revascularization. Interestingly, there was a trend towards higher risk of death from noncardiovascular causes in patients treated with colchicine, although statistically insignificant [51].

Since colchicine proved its efficacy in LoDoCo and LoDoCo2 trials on patients with stable coronary disease, its anti-inflammatory effect in unstable patients was questioned. At the same time, colchicine was tested in patients with recent myocardial infarction, in COLCOT (Colchicine Cardiovascular Outcomes Trial) trial [56]. Study population were patients with recent myocardial infarction (within 30 days after the event). Patients from one group were randomized to low-dose colchicine (0.5 mg once daily), while the other group received placebo. Primary outcome was broader than in LoDoCo2 trial, and included composite of death from cardiovascular causes, resuscitated cardiac arrest, myocardial infarction, stroke, or urgent hospitalization for angina leading to coronary revascularization [56].

A total of 4745 patients with recent myocardial infarction were enrolled. After a median follow-up of almost two years (22.6 months), adverse cardiovascular events occurred less frequently in colchicine-group compared to placebo-group (5.5% vs. 7.1%, respectively). This difference translated into a relative risk reduction of 23% with the administration of colchicine (HR 0.77, 95% CI 0.61–0.96, *p* = 0.02). Analyzing the components of primary outcome, colchicine significantly reduced the risk of stroke (by 74%) and urgent coronary revascularization (by 50%), with no significant impact on cardiovascular mortality, resuscitated cardiac arrest, and myocardial infarction [56]. This additive value of colchicine was recorded despite adequate background therapy, which in 98–99% of patients included aspirin, antiplatelet agent, and statin.

The most common adverse events were gastrointestinal, which is in line with the known safety profile of colchicine [57]. It should be mentioned that serious adverse events, such as infection and pneumonia, were more frequent in colchicine-group than in placebo-arm (2.2% vs. 1.6%, and 0.9% vs. 0.4%, respectively). Two possible explanations for differences in infection emerged: one might be due to the play of chance, and other might be the immunologic response to colchicine [56].

*Post hoc* analysis of COLCOT trial pointed to the time-dependent effect of colchicine initiation [58]. If the colchicine was initiated within 3 days after the myocardial infarction, MACE was reduced by 48% compared to placebo. On the other hand, if the administration of colchicine started later, between the 4th and 7th day after myocardial infarction, there was no difference in primary outcome between colchicine- and placebo-arm. Therefore, it seems that the timing of anti-inflammatory therapy initiation is important for patients after myocardial infarction.

Another study that included patients after acute myocardial infarction tested the ability of colchicine to reduce the levels of CRP [59]. This was a pilot-study, LoDoCo-MI (Low Dose Colchicine after Myocardial Infarction) which included 237 patients following acute myocardial infarction. As in previous trials, patients were randomized to low-dose colchicine (0.5 mg daily), or placebo. Anti-inflammatory effect of colchicine in an acute setting was assessed by CRP levels after 30 days. Unexpectedly, low-dose colchicine was not associated with higher likelihood of achieving a CRP level <2 mg/L. Additionally, the average absolute reduction in CRP levels was similar between colchicine-group (–4.3 mg/L) and placebo-group (–3.3 mg/L) [59].

This was in line with the next clinical trial, COPS trial, which included 795 patients with acute coronary syndrome, randomized to colchicine or placebo [60]. Low dose colchicine was administered 0.5 mg twice daily in the first month and 0.5 mg daily during the following 11 months. Primary outcome was MACE, a composite of all-cause mortality, acute coronary syndrome (ACS), ischemia-driven (unplanned) urgent revascularization, and noncardioembolic ischemic stroke. After 1 year of follow-up there was no difference in MACE, with 24 events in colchicine-group and 38 events in placebo-arm (*p* = 0.09). However, there was a higher rate of mortality with colchicine, particularly all-cause mortality (8 vs. 1, *p* = 0.017), and noncardiovascular death (5 vs. 0, *p* = 0.024) [60].

These findings were quite surprising. It is well known that patients in acute settings, such as acute coronary syndrome, have higher levels of inflammation than patients with stable coronary artery disease. Therefore, it is expected colchicine to show a pronounced anti-inflammatory effect in acute settings. Explanation for the discrepancy might be in colchicine’s mechanism of action.

Namely, the primary effect of colchicine is the inhibition of microtubules, which impairs the mobility and activation of inflammatory cells [61]. Additionally, microtubules constitute the NLRP3 inflammasome, so colchicine can indirectly inhibit this important inflammation pathway [62]. As a result, a smaller amount of IL-1β is generated from its inactive form, reflecting the reduced secretion of IL-6.

Some authors report that NLRP3 inflammasome might have a minor role in the acute coronary syndrome since its low expression in myocardium [63], which could partially explain negative results of colchicine in acute coronary syndrome. However, this hypothesis remains elusive and needs further clarification since other authors claim the opposite — that NLRP3 inflammasome has an important role in acute-setting inflammation [64, 65].

## 10. Allopurinol: Negative Results from ALL-HEART Study 

Rationale behind the allopurinol testing in coronary artery disease resides on its anti-oxidative and anti-inflammatory effect, and also the association of hyperuricemia with increased cardiovascular risk [66, 67]. Allopurinol is a purine analogue, an inhibitor of xanthine oxidase which acts as urate-lowering therapy. It is indicated in the therapy of gout, prevention of tumour lysis syndrome, and prevention of recurrent calcium nephrolithiasis in patients with hyperuricosuria [68, 69]. Normally, xanthine oxidase generates oxidative free radicals, which are associated with endothelial dysfunction and inflammation [70]. Therefore, repurposing allopurinol in patients with coronary artery disease might be an additional method for silencing inflammation and improving outcomes of these patients.

Just recently the results of a long awaited ALL-HEART study on effects of allopurinol in patients with ischemic heart disease but no history of gout were published [71]. Study included almost 6000 patients who were aged ≥60 years, with a history of ischemic heart disease (myocardial infarction, angina, or other evidence of ischaemic heart disease, such as a positive coronary angiogram). Patients with a history of gout were excluded. All patients continued with usual care, while one group was randomly assigned to oral allopurinol (up-titrated to a dose of 600 mg daily, or 300 mg daily in case of moderate renal impairment at baseline). During mean follow-up of 4.8 years, MACE was recorded as composite of non-fatal myocardial infarction, non-fatal stroke, or cardiovascular death.

After the follow-up was completed, there was no difference in the composite cardiovascular outcome, observed in 11% of patients from allopurinol-group, and 11.3% patients in the usual care group (HR 1.04, 95% CI 0.89–1.21, *p* = 0.65) [71].

There are several explanations for negative results of ALL-HEART study. First, expecting the translation of xanthine oxidase inhibition into hard clinical endpoints might be overestimated. Selective inhibition of pro-oxidative capability of xanthine oxidase can be detected on cellular and biochemical levels, but it is less likely to make a difference in composite cardiovascular outcome [72]. Second, 57.4% participants from allopurinol-group stopped taking this medication by the end of the study, most commonly due to adverse events or participants’ preference. On the other hand, the majority of patient already had optimal medicament therapy, including statins, renin-angiotensin-aldosterone system inhibitors, and beta-blockers, where some on them already had indirect anti–inflammatory and anti-oxidative effects [73].

In contrast to LoDoCo and LoDoCo2 studies, ALL-HEART population was much older, at an average 71 years. And finally, patients were not selected based on their oxidative stress. Unfortunately, there is no known universal marker of oxidative stress that could help identify patients with higher oxidative burden, and help guide antioxidative therapy.

## 11. Other Cardiovascular Drugs with Secondary Anti-Inflammatory Effects

Many well-known and established cardiovascular drugs, such as trimetazidine, nebivolol, zofenopril, rosuvastatin, and omega-3 polyunsaturated acids, exert pleiotropic anti-oxidant and secondary anti-inflammatory effects. It is plausible that the cardioprotective effect of these agents is augmented by their anti-oxidative and anti-inflammatory properties, clearly demonstrated in preclinical studies.

Trimetazidine is an anti-ischemic drug widely used in patients with coronary artery disease. Antianginal effect of this drug is achieved via modulation of cardiac metabolism. Trimetazidine inhibits mitochondrial enzyme (long-chain mitochondrial 3-ketoacyl coenzyme A thiolase enzyme) involved in β-oxidation of free fatty acids. As a result, cardiac metabolism is shifted from utilization of free fatty acids as a primary source of energy, to stimulation of glucose oxidation. That way the production of adenosine triphosphate (ATP) is more efficient in ischemic conditions [74]. While modulating cardiac metabolism, trimetazidine has no significant effect on hemodynamic parameters, such as coronary flow, contractility, heart rate, and blood pressure, providing additive value to standard antianginal therapy.

Anti-oxidative effects of trimetazidine result in reduced release of proinflammatory cytokines from macrophages stimulated by oxidative stress, such as tumor necrosis factor-α (TNF-α), IL-1β, and IL-6 [75, 76]. Therefore, it is possible that additional anti-oxidative and secondary, anti-inflammatory effects act synergistically in patients with coronary artery disease.

Nebivolol is a cardioselective, beta-1 adrenergic receptor antagonist, which exerts a positive effect on endothelial function. This beta-blocker additionally stimulates nitric oxide (NO) synthase, which leads to NO-mediated vasodilatation. Its dual antihypertensive properties (β-blockade and NO-mediated vasodilatation) singled out nebivolol as one of the preferred hypertensive agents [77]. Additionally, nebivolol has strong antioxidant activity which provides multiple beneficial effects on cardiovascular health. Interestingly, patients with essential hypertension treated with nebivolol had significantly lower reduction of basal and stimulated NO induced by oxidative stress compared to patients receiving atenolol [78].

Another powerful cardiovascular drug, zofenopril, exerts significant antioxidative properties. This angiotensin-converting enzyme (ACE) inhibitor can stimulate NO production, decrease the progression of atherosclerosis, and inhibit the expression adhesion molecules on endothelial cells by reducing reactive oxygen species [79, 80]. Taken together, the cardioprotective role of zofenopril is reinforced by its antioxidative properties.

Another interesting anti-inflammatory approach includes nutritional interventions with omega-3 polyunsaturated fatty acids (n-3 PUFA), such as eicosapentaenoic acid (EPA) and docosahexaenoic acid (DHA). These n-3 fatty acids are found in oily fish and fish oil supplements, and exert diverse anti-inflammatory effects, attractive for patients with coronary artery disease [81]. EPA and DHA act on many steps of coronary atherosclerosis; n-3 PUFA can reduce the expression of adhesion molecules on endothelial cells, inhibit leukocyte-endothelial cell adhesion, and reduce the production of inflammatory cytokines [82].

Positive preclinical results translated into firm clinical evidence on efficacy of n-3 PUFA in patients with cardiovascular disease. Recently published REDUCE-IT trial enrolled 8179 patients, randomly assigned to treatment with icosapent ethyl, a highly purified and stable EPA ethyl ester, or placebo [83]. Included patients had either established cardiovascular disease, or diabetes with other risk factors. All participants had hypertriglyceridemia (fasting triglyceride level of 1.52–5.63 mmol/L), and LDL-cholesterol level of 1.06–2.59 mmol/L despite statin therapy. Patients were receiving icosapent ethyl (2 g, twice daily), or placebo.

The efficacy was evaluated by a composite of cardiovascular death, nonfatal myocardial infarction, nonfatal stroke, coronary revascularization, or unstable angina. After a median follow-up of 4.9 years, supplementation with icosapent ethyl led to 25% risk reduction of primary outcome compared to placebo-group. Namely, adverse cardiovascular events were recorded in 17.2% patients in the icosapent ethyl-group, and in 22.0% of the patients in the placebo-arm [83].

Recently, *post hoc* analysis of the REDUCE-IT trial reinforced the position of icosapent ethyl supplementation in secondary cardiovascular prevention [84]. A total of 3693 patients from the REDUCE-IT trial had previous myocardial infarction, in whom icosapent ethyl significantly reduced the risk of adverse cardiovascular events from 26.1% to 20.2% compared to placebo. In this subgroup of patients, icosapent ethyl reduced the risk of cardiovascular death by 30%, along with 20% relative risk reduction in all-cause mortality.

## 12. Conclusions

Since atherosclerosis may be regarded as an inflammatory response to injury and endothelial dysfunction, targeting inflammation could be the novel therapeutic approach in treating patients with coronary artery disease. Current cardiovascular research is focused primarily on finding and targeting molecular pathways and inflammatory markers underlying atherosclerosis that would be clinically effective in improving outcomes in cardiovascular patients. Experimental and clinical research point to the NLRP3 inflammasome/IL-1β/IL-6/CRP pathway, important for targeted and non-targeted drug therapy in coronary artery disease. So far, clinical results have given modest results with inhibiting only one point or one molecule in this pathway. Therefore, the introduction of an agent that can inhibit several stages in the NLRP3 inflammasome/IL-1β/IL-6/CRP pathway might be a more efficient strategy to improve prevention and therapy in patients with coronary artery disease.
